# Cellular Size, Gap Junctions, and Sodium Channel Properties Govern Developmental Changes in Cardiac Conduction

**DOI:** 10.3389/fphys.2021.731025

**Published:** 2021-10-25

**Authors:** Madison B. Nowak, Rengasayee Veeraraghavan, Steven Poelzing, Seth H. Weinberg

**Affiliations:** ^1^Department of Biomedical Engineering, The Ohio State University, Columbus, OH, United States; ^2^The Ohio State University Wexner Medical Center, Davis Heart and Lung Research Institute, Columbus, OH, United States; ^3^Department of Biomedical Engineering and Mechanics, Virginia Polytechnic Institute and State University, Blacksburg, VA, United States; ^4^Virginia Polytechnic Institute and State University, Fralin Biomedical Research Institute at Virginia Tech Carilion, Roanoke, VA, United States

**Keywords:** cardiac electrophysiology, computational models, intercalated disc, development, cardiac conduction

## Abstract

Electrical conduction in cardiac ventricular tissue is regulated via sodium (Na^+^) channels and gap junctions (GJs). We and others have recently shown that Na^+^channels preferentially localize at the site of cell-cell junctions, the intercalated disc (ID), in adult cardiac tissue, facilitating coupling via the formation of intercellular Na^+^nanodomains, also termed ephaptic coupling (EpC). Several properties governing EpC vary with age, including Na^+^channel and GJ expression and distribution and cell size. Prior work has shown that neonatal cardiomyocytes have immature IDs with Na^+^channels and GJs diffusively distributed throughout the sarcolemma, while adult cells have mature IDs with preferentially localized Na^+^channels and GJs. In this study, we perform an *in silico* investigation of key age-dependent properties to determine developmental regulation of cardiac conduction. Simulations predict that conduction velocity (CV) biphasically depends on cell size, depending on the strength of GJ coupling. Total cell Na^+^channel conductance is predictive of CV in cardiac tissue with high GJ coupling, but not correlated with CV for low GJ coupling. We find that ephaptic effects are greatest for larger cells with low GJ coupling typically associated with intermediate developmental stages. Finally, simulations illustrate how variability in cellular properties during different developmental stages can result in a range of possible CV values, with a narrow range for both neonatal and adult myocardium but a much wider range for an intermediate developmental stage. Thus, we find that developmental changes predict associated changes in cardiac conduction.

## 1. Introduction

It is well-established that conduction in cardiac tissue is regulated by ionic currents and gap junction (GJ) coupling (Shaw and Rudy, 1997; Kucera et al., 2002). In ventricular tissue, the voltage-gated sodium (Na^+^) channel, Na_v_1.5, is primarily responsible for generating the depolarizing Na^+^current (*I*_*Na*_), and connexin43 (Cx43) is the primary GJ protein, facilitating the passive current flow between adjacent cells (Veeraraghavan et al., 2014), both of which mediate conduction. Altering either *I*_*Na*_or GJ coupling can lead to changes in conduction and ultimately increase the risk of arrhythmias (Quan and Rudy, 1990; Shaw and Rudy, 1997; Rohr et al., 1998).

We (Veeraraghavan et al., 2015; Veeraraghavan and Gourdie, 2016; Mezache et al., 2020) and others (Kucera et al., 2002; Rhett et al., 2012; Agullo-Pascual et al., 2014; Leo-Macias et al., 2016) have shown that Na_v_1.5channels preferentially localize at the intercalated disc (ID), the area of cell-cell junctions in cardiac tissue. Multiple *in silico* studies have hypothesized that *I*_*Na*_at the ID can be altered via Na^+^nanodomain signaling at the intercellular cleft space (Kucera et al., 2002; Sperelakis, 2002; Mori et al., 2008; Lin and Keener, 2010; Wei et al., 2016; Tveito et al., 2017; Weinberg, 2017; Hichri et al., 2018; Jæger et al., 2019; Wei and Tolkacheva, 2020). This, in turn, can modulate cell-cell coupling through a mechanism known as ephaptic coupling (EpC). In this paper we consider two primary effects of EpC: electrical field effects and Na^+^depletion in the intercellular cleft (i.e., the narrow extracellular space between electrically coupled cells at the ID). To elaborate briefly, EpC is governed by the following: Na^+^influx during the action potential upstroke in an “upstream” or pre-junctional depolarizing cell during a propagating electrical wave decreases the electrical potential within the intercellular cleft. This reduction of the potential within the intercellular cleft then depolarizes the “downstream” or post-junctional apposing cell from the extracellular, rather than the intracellular, side of the cell membrane. Additionally, Na^+^influx reduces the Na^+^concentration within the intercellular cleft, which governs the flux of the Na^+^channels at the ID in both cells. The width or volume of the intercellular cleft space is one of the key properties governing the magnitude of these effects. When the intercellular cleft is narrow, both the elevated transmembrane potential (*V*_*m*_) and locally depleted Na^+^concentration within the intercellular cleft reduce the Na^+^current driving force and, therefore, the Na^+^current. This reduction in *I*_*Na*_has been termed “self-attenuation” and has been shown to slow conduction velocity (CV) (Kucera et al., 2002; Sperelakis, 2002; George et al., 2016; Hichri et al., 2018).

Several key properties governing EpC and conduction overall are known to change during development: Cells in neonatal myocardium do not have fully formed IDs, and Na^+^channels and GJs are distributed diffusively throughout the sarcolemma (Fromaget et al., 1992; Vreeker et al., 2014). Consistent with reduced Na^+^channel expression, it has been shown that pediatric cardiomyocytes produce a reduced *I*_*Na*_, compared to adult cardiomyocytes (Cai et al., 2011; Cordeiro et al., 2013). Cx43 is essentially undetectable until 23 weeks *in utero* and remain randomly distributed on the sarcolemma in neonatal cardiomyocytes (Peters et al., 1994; Hirschy et al., 2006; Vreeker et al., 2014; Swift et al., 2020). Vreeker et al. showed that Cx43 tends to relocate to the lateral membrane around 5 months postnatal and does not begin to preferentially localize at the ID until around 2.5–5 years old, with full preferential localization occurring at roughly 7 years of age (Vreeker et al., 2014). Na_v_1.5channels, however, reside on the lateral membrane in neonatal cardiomyocytes and begin to begin to preferentially localize at the ID around 5 months postnatal, much earlier than Cx43 (Harrell et al., 2007; Vreeker et al., 2014). Additionally, studies have shown that adult cardiomyocytes are larger than neonatal cardiomyocytes (Cordeiro et al., 2013; Vreeker et al., 2014; Swift et al., 2020). This is especially important given that cell size broadly influences all electrical activity in the cell by altering surface area, cell volume, membrane capacitance, ion channel expression, etc. (Kato et al., 1996; Spach et al., 2000).

We hypothesize that the developmental-associated increase in both Na^+^and GJ current will increase CV from the neonatal stage to the adult and that this regulation will be influenced by the relative strength of EpC at different developmental stages. In the paper, we perform a wide parameter investigation, varying age-associated parameters including gap junctional conductance (*f*_*gap*_), cell size (*S*), Na^+^channel density (ρ_*Na*_), and Na^+^channel ID localization (*ID*_*Na*_) and measure CV in simulated cardiac tissue. To our knowledge, no studies have investigated the interdependence of these parameters on conduction within a health myocardium during development from neonatal to adult tissue. While one study investigated the changes on impulse conduction in the canine myocardium, it was limited to a comparison of 8 week old postnatal to adult purkinje fibers (Rosen et al., 1981). Thus, in our study, we investigate conduction through ventricular tissue in a range of developmental stages and conditions. We find that CV has a biphasic dependence on cell size, in a manner that depends on GJ coupling. In addition, we find that ephaptic effects play a larger role in conduction for larger cells with low GJ coupling. Interestingly, simulations predict that variability in cellular properties in intermediate developmental stage between neonatal cardiomyocytes and adult cardiomyocytes can lead to a wide range of possible CV values, but this range narrows to a normal range with adult tissue-associated parameters.

## 2. Methods

**Figure 1:**
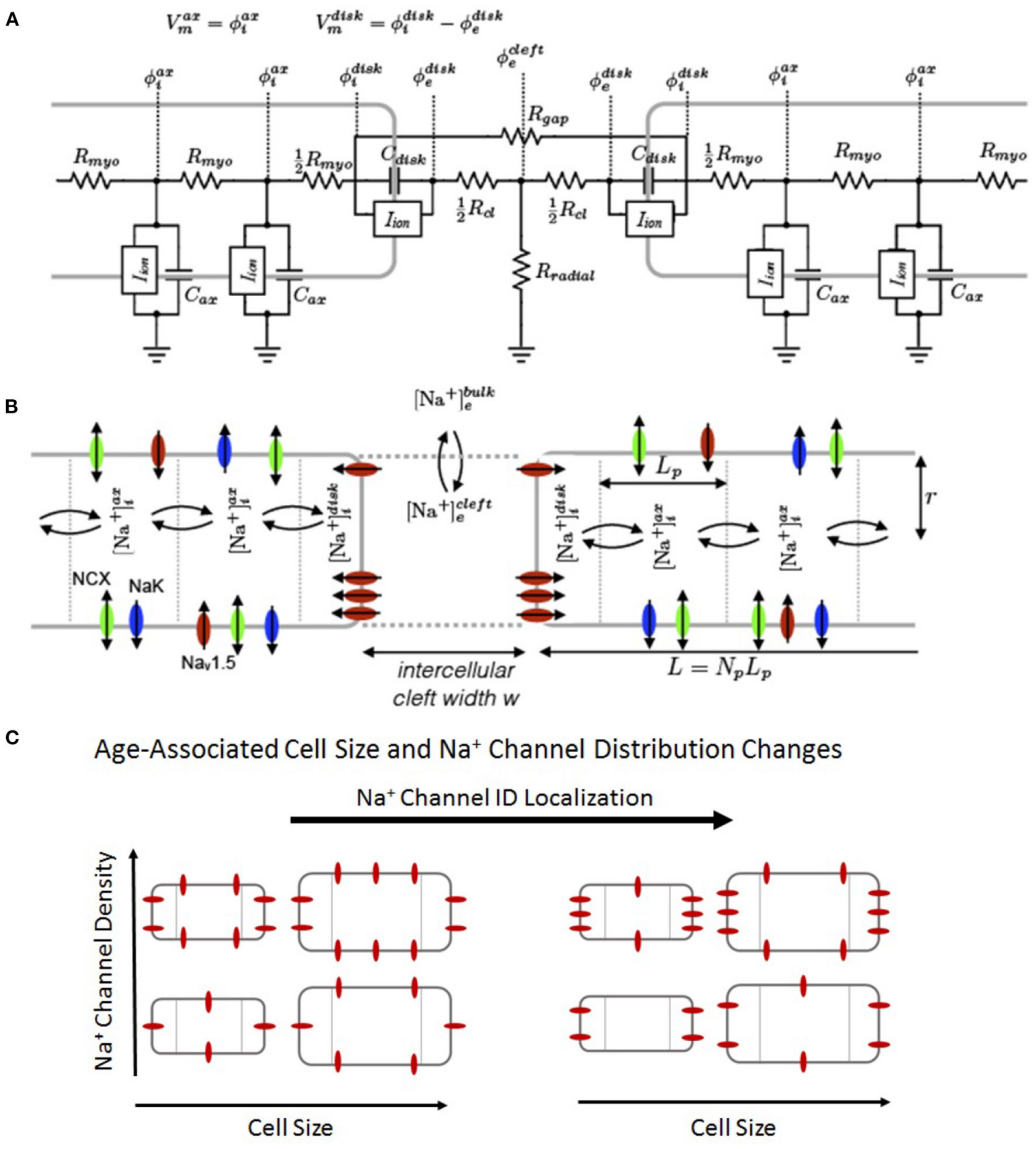
Schematic of the computational model. **(A)** Electric circuit representation of coupled myocytes. Intracellular nodes are coupled via a myoplasmic resistance (*R*_*myo*_). End nodes are coupled via a gap junctional resistance (*R*_*gap*_). Extracellular potentials at the disc and intercellular cleft ($\phi_{e}^{disc}$ and $\phi_{e}^{cleft}$, respectively) are governed by a T-shaped network of two axial resistances in the intercellular cleft (*R*_*cl*_) and one radial resistance (*R*_*radial*_). **(B)** Na^+^concentration in diffusively coupled compartments, including intracellular Na^+^in the axial and disc compartments (${\left[Na^{+}\right]}_{i}^{ax}$ and ${\left[Na^{+}\right]}_{i}^{disc}$) and extracellular Na^+^in the intercellular cleft and bulk spaces (${\left[Na^{+}\right]}_{e}^{cleft}$ and ${\left[Na^{+}\right]}_{e}^{bulk}$). **(C)** Representation of age-associated change in model parameters, including changes in cell size (*S*), Na^+^ channel density (ρ_*Na*_), and Na^+^channel ID localization (*ID*_*Na*_).

Full details of the computational model are provided in Supplementary Material. Briefly, we simulate a 50-cell cable of guinea pig ventricular myocytes (Livshitz and Rudy, 2007) that incorporates a Markov chain model for the wild-type (WT) Na^+^channel (Clancy et al., 2002), shown in Figure 1. We note that a Markov chain formulation for *I*_*Na*_ was not necessary to reproduce our results, as prior studies have shown EpC effects are reproduced by Hodgkin-Huxley Na^+^channel models (Kucera et al., 2002; Weinberg, 2017; Moise et al., 2021), but rather is used to facilitate an appropriate comparison with our prior work simulating Na_v_1.5gain-of-function mutations (Nowak et al., 2021), which were also modeled with a Markov chain. As in our previous work (Greer-Short et al., 2017; Weinberg, 2017; Nowak et al., 2020, 2021) and work performed by others (Kucera et al., 2002), we account for non-uniform Na^+^subcellular localization by spatially discretizing each cell into two ID membrane patches at the ends of the cell and axial membrane patches along the length of the cell. The number of axial patches varied with the size of the cell, as described below, with each axial patch fixed in length (*L*_*p*_ = 10 μm).

Cells are coupled via GJs and EpC: gap junctional coupling is represented via gap junctional conductances coupling ID nodes of adjacent cells (*g*_*gap*_, represented by resistor *R*_*gap*_ in the electrical circuit). EpC is represented by a T-shaped junction of two intercellular cleft resistances (*R*_*cl*_) and a radial bulk (*R*_*radial*_), which are proportional and inversely proportional to intercellular cleft width *w*, respectively. Intracellular nodes are coupled with a myoplasmic resistance (*R*_*myo*_) (Figure 1A). The nominal value for *g*_*gap*_ is defined as $g_{gap}^{0}=1266nS$, and changes in GJ coupling are accounted for by adjusting the GJ scaling factor *f*_*gap*_ (between 0 and 1), such that $g_{gap}=f_{gap}g_{gap}^{0}$.

A wide range of macroscopic GJ conductance values have been measured experimentally, with values ranging from the low 10s of nanosiemens up to approximately 2,000 nS (Weingart, 1986; Wittenberg et al., 1986; White et al., 1990; Moreno et al., 1994; Kwak and Jongsma, 1996; Verheule et al., 1997; Kucera et al., 2002; Valiunas et al., 2002; Desplantez et al., 2007; McCain et al., 2012; Nielsen et al., 2012). While studies have shown increases in Cx43 expression with age, to our knowledge, no studies have quantified GJ conductances throughout development. Therefore, we performed simulations with GJ conductance spanning from the low to high end of physiological measurements and hypothesized that such a range is qualitatively similar to different developmental stages and consistent with Cx43 expression changes. Importantly, the values chosen were consistent with experimental measurements of conduction for neonatal and adult myocardium (Rosen et al., 1981; George et al., 2019; King et al., 2021). We note that age-associated changes in GJ localization are represented as changes in GJ coupling (i.e., changes in *f*_*gap*_), as all GJs are located at cell ends in the one-dimensional tissue model.

We account for dynamic [Na^+^] in three spaces: (i) the ID (${\left[\text{N}{\text{a}}^{+}\right]}_{i}^{disc}$), mediated by $I_{Na}^{disc}$ and intracellular diffusion, (ii) the intercellular cleft (${\left[\text{N}{\text{a}}^{+}\right]}_{e}^{cleft}$), with volume proportional to *w* and mediated by ID Na^+^current $I_{Na}^{disc}$ and passive diffusion with the bulk extracellular space, and (iii) the axial intracellular space (${\left[\text{N}{\text{a}}^{+}\right]}_{i}^{ax}$), mediated by axial Na^+^current $I_{Na}^{ax}$ and intracellular diffusion. The cable was paced at one end with a specified basic cycle length (BCL). Unless otherwise stated, for all simulations, we utilize a *BCL* = 500 ms or a pacing rate of 2 Hz, which is normal pacing for the guinea pig model that is utilized for this study.

In addition to GJ coupling, we perform simulations in which we adjust several key age-associated properties: (1) We vary Na^+^channel localization at the ID (*ID*_*Na*_) between 0.1 and 1, to account for Na^+^channel redistribution, where *ID*_*Na*_ = 1 represents 100% or all Na^+^ channels localized at the ID. (2) We define and vary a cell size scaling factor (*S*) between 0.2 and 1 to account for cell size growth, where 100% cell size represents an adult cell. The cell geometry is assumed to be cylindrical, with radius *r* and length *L*, defined as *r* = *Sr*_0_ and *L* = *n*_*p*_*L*_*p*_, where the nominal adult radius *r*_0_ = 11 μm, the number of axial patches $n_{p}=Sn_{p}^{0}$, and the maximum axial membrane patches $n_{p}^{0}=10$. Note, we only consider values of *S*such that *n*_*p*_ is a whole number, and that nominal adult cell length $L_{0}=n_{p}^{0}L_{p}=100\;\mu \text{m}$. Additionally, note that since *S* scales both cell length and radius, cell membrane surface area is scaled by *S*^2^, e.g., *S* = 0.4 corresponds with surface area scaled by a factor of 0.16. (3) We also vary the Na^+^channel density (ρ_*Na*_) between 0.2 and 1 to account for age-associated changes in Na^+^channel expression, where 100% ρ_*Na*_represents full expression of Na^+^channels on the cell membrane. The total cellular Na^+^conductance is proportional to both ρ_*Na*_and total cell surface area, such that we can define a normalized total Na^+^channel conductance ($G_{Na}=\rho_{Na}S^{2}$, also between 0 and 1), where the total Na^+^channel conductance (in physical units) is equal to *G*_*Na*_, scaled by the nominal total Na^+^channel conductance ($G_{Na}^{0}=21.78\text{ mS}/\text{c}{\text{m}}^{2}$). Thus, for ρ_*Na*_and *S*of 100%, total Na^+^ channel conductance is $G_{Na}^{0}$. (4) Finally, we vary the intercellular cleft width (*w*) from 10 to 40 nm, consistent with intercellular cleft width ranges measured at the ID in our previous work (Veeraraghavan and Gourdie, 2016; Greer-Short et al., 2017; Nowak et al., 2020, 2021).

## 3. Results

### 3.1. Ephaptic Effects Are Enhanced for Larger Cells With Low Gap Junctional Coupling

**Figure 2:**
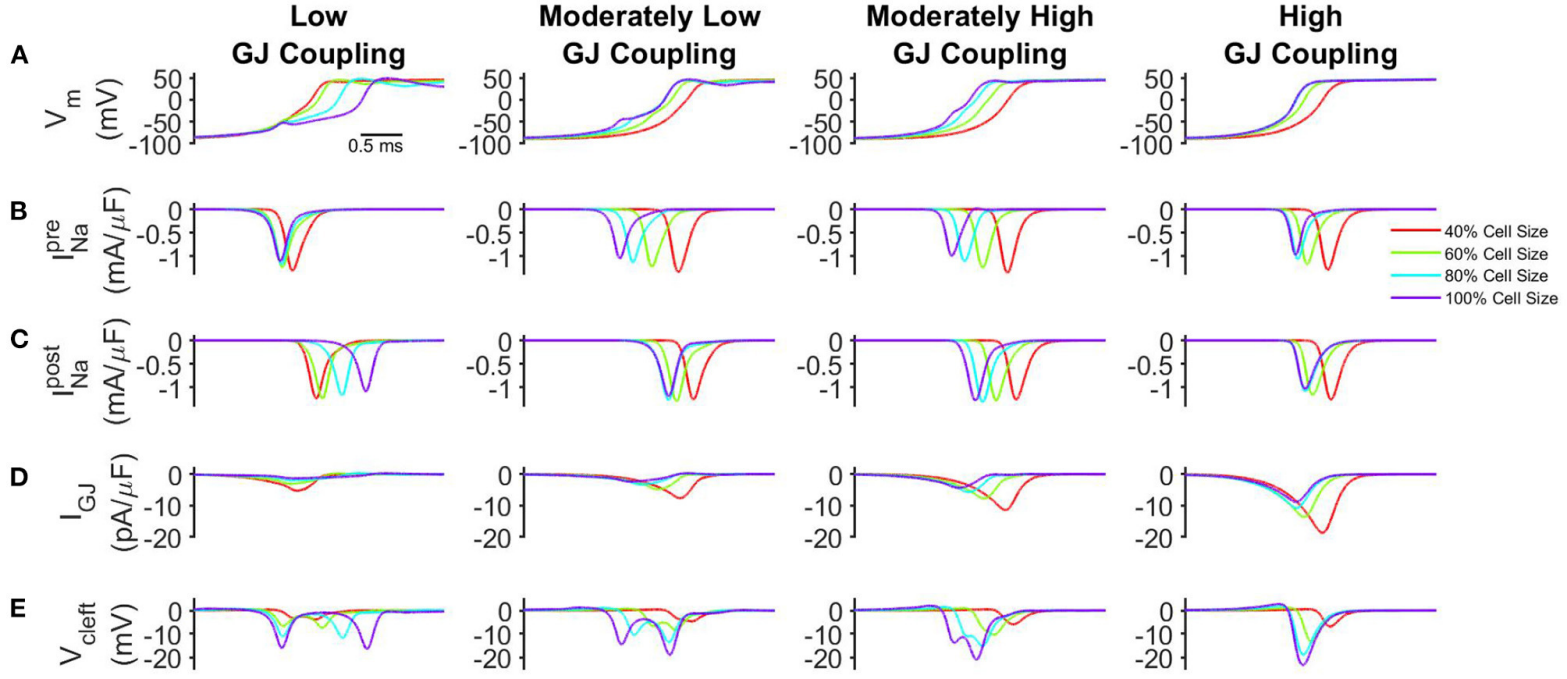
Conduction velocity (CV) depends on cell size and GJ coupling. **(A)** Transmembrane voltage of the post-junctional node of the ID (*V*_*m*_), **(B)** Na^+^current at the pre-junctional node of the ID ($I_{Na}^{pre}$), **(C)** Na^+^current at the post-junctional node of the ID ($I_{Na}^{post}$), **(D)** GJ current at the ID (*I*_*GJ*_), and **(E)** cleft voltage (*V*_*cleft*_) are shown in tissue for low (50.6 nS), moderately low (101 nS), moderately high (253 nS), and high (1,266 nS) GJ coupling. For clarity, traces are shown for the same spatial point of 1.2 mm from the pacing site. Parameters: *ID*_*Na*_= 50%, ρ_*Na*_= 100%, *w* = 20*nm*.

Motivated by findings that both GJ coupling (Peters et al., 1994; Hirschy et al., 2006; Vreeker et al., 2014; Swift et al., 2020) and cell size (Kato et al., 1996; Spach et al., 2000; Cordeiro et al., 2013; Vreeker et al., 2014) increase with age, we first investigate how changes in cell size and GJ coupling influence conduction. We first consider moderate localization of Na^+^channels at the ID (*ID*_*Na*_ = 50%), high whole cell Na^+^channel density (ρ_*Na*_ = 100%), and a nominal intercellular cleft width (*w* = 20 nm). In Figure 2, the time series for transmembrane voltage (*V*_*m*_), pre- and post-junctional Na^+^current ($I_{Na}^{pre}$and $I_{Na}^{post}$, respectively), GJ current (*I*_*GJ*_), and the cleft voltage (*V*_*cleft*_) are shown during the action potential upstroke at the same spatial location within the cardiac tissue for varying cell size and GJ coupling.

For low GJ coupling (Figure 2, *left*), the slowed action potential upstroke for the large cell size indicates that conduction slows as cell size increases. For tissue with small cell size, a large pre-junctional current $I_{Na}^{pre}$rapidly activates the post-junctional current $I_{Na}^{post}$ (Figures 2B,C, red), and the smaller membrane surface area results in a relatively larger GJ current density *I*_*GJ*_ (Figure 2D, red), compared with larger cell size. In contrast, for tissue with larger cell size, GJ current density is reduced (Figure 2D, purple), and further cleft hyperpolarization is enhanced, i.e., *V*_*cleft*_is more negatively polarized (Figure 2E, purple). This enhanced EpC effect ultimately drives the self-attenuation mechanism, in which $I_{Na}^{post}$driving force is reduced. Collectively, these effects result in slower conduction for tissue with larger cell size.

For moderately low and moderately high GJ coupling (Figure 2, *center left* and *center right*), these EpC effects are reduced; however, cleft hyperpolarization is still enhanced for larger cell sizes (Figures 2B–E). For high GJ coupling (Figure 2, *right*), the faster action potential upstroke for large cell size indicates that conduction is enhanced as cell size increases. In this case, *I*_*GJ*_is sufficiently large, such that the self-attenuation effects on post-junctional Na^+^current are counterbalanced, in a manner that conduction is faster for tissue with larger cell size. Additionally, for larger cell size, there are fewer cell-cell junctions in a given length of tissue, which also results in overall faster conduction.

### 3.2. Conduction Velocity Depends on Key Cellular and Tissue Properties

**Figure 3:**
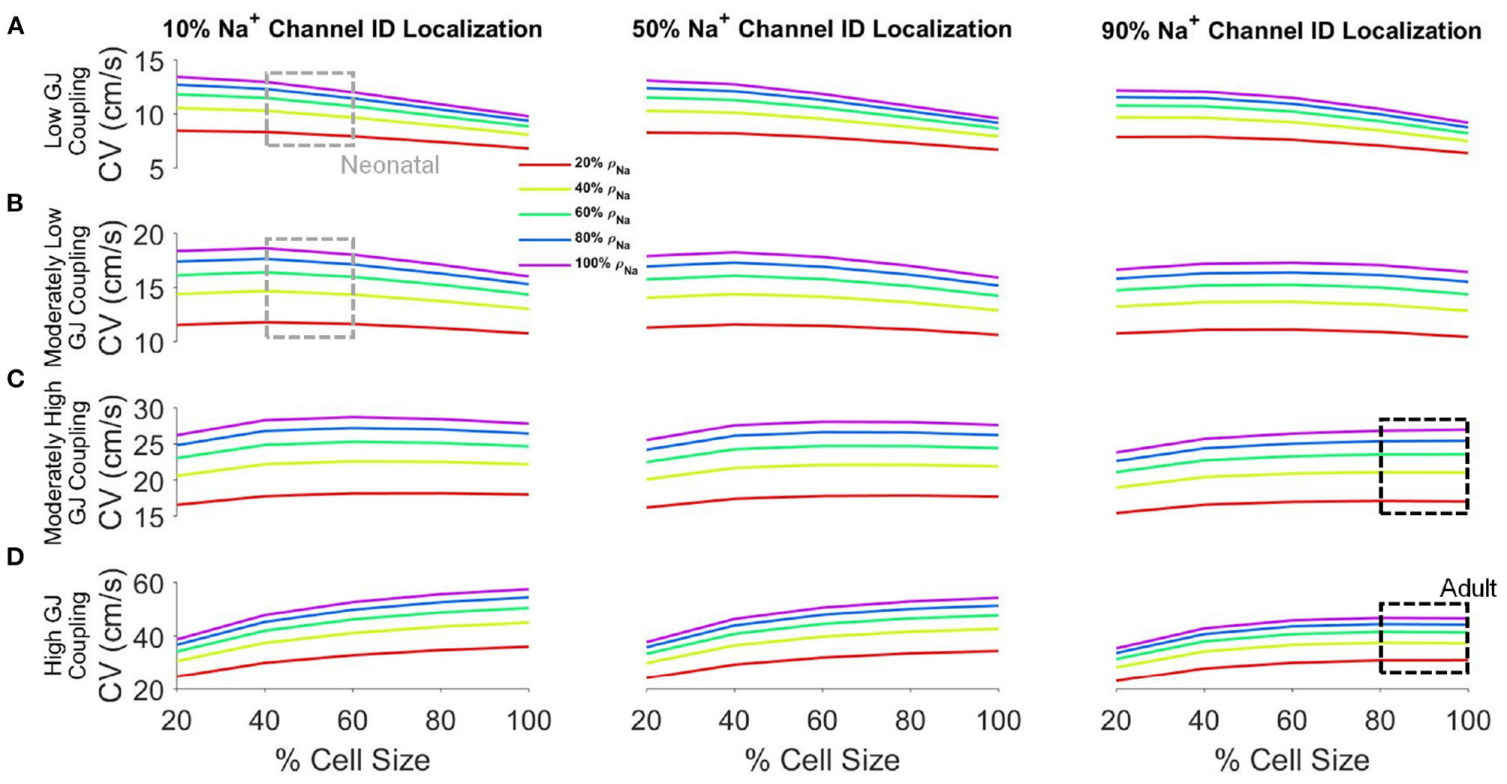
Conduction velocity (CV) depends on key cellular and tissue properties. CV is shown as a function of cell size for different values of Na^+^channel densities (ρ_*Na*_) for low **(A)**, moderately low **(B)**, moderately high **(C)**, and high **(D)** GJ coupling and 10% (*left*), 50% (*middle*), and 90% (*right*) Na^+^channel ID localization (*ID*_*Na*_). Parameters: Cleft width *w* = 20*nm*. Parameter regimes associated with neonatal (gray boxes) and adult (black boxes) tissue are highlighted.

We next more broadly investigated the interdependence between cell size, whole cell Na^+^channel density (ρ_*Na*_), Na^+^channel ID localization (*ID*_*Na*_), and GJ coupling on CV (Figure 3). We consider a wide range of parameters to assess the age-associated changes on CV. For all cases investigated, as expected, CV consistently increases with both increasing GJ coupling and ρ_*Na*_. For low GJ coupling (Figure 3A), as illustrated above, CV decreases as cell size increases, due to both the reduced GJ current density and the self-attenuation mechanism previously stated. Increased Na^+^channel preferential localization at the ID tends to moderately increase CV for larger cell sizes due to increased EpC, which increases conduction for low GJ coupling.

In contrast, for high GJ coupling (Figure 3D), CV increases as cell size increases due to fewer GJs per unit length, as described above. As *ID*_*Na*_increases, CV decreases moderately, more so for larger cell sizes, again due to increased EpC, which decreases conduction for high GJ coupling. From low to high GJ coupling, the CV-cell size relationship transitions from decreasing to increasing, such that for moderate GJ coupling strengths, CV exhibits a biphasic relationship with cell size (Figures 3B,C). However, for most conditions, CV only moderately varies across the wide range of cell sizes, which suggests GJ and EpC effects that depend on cell size are fairly balanced for these conditions. Increased Na^+^channel ID localization has only small effects, tending to increase CV for the moderately low GJ coupling cases.

We indicate two key regions of interest: the neonatal regime and the adult regime. The neonatal regime is indicated by gray boxes and represents the range of parameters associated with neonatal myocardium: small cell size, low Na^+^channel ID localization, and low to moderately low GJ coupling (Peters et al., 1994; Kato et al., 1996; Spach et al., 2000; Vreeker et al., 2014). The adult regime is indicated by black boxes and represents the range of parameters associated with adult myocardium: large cell size, high preferential Na^+^channel localization at the ID, and moderately high to high GJ coupling. In the neonatal regimes, CV is slow, consistent with the reduced GJ coupling, and further is sensitive to changes in cell size. In contrast, in the adult regime, CV is faster, consistent with the higher GJ coupling, and additionally does not depend on cell size. In both regimes, CV is highly sensitive to changes in Na^+^channel density. Collectively, this suggests that developmental changes result in conduction that is more robust to changes in cell size. Additionally, in Supplementary Material, we perform a similar broad investigation for a bradycardic pacing rate (1 Hz, or BCL of 1,000 ms), and we found that CV has a nearly identical relationship with cell size, Na^+^channel density and localization, and GJ coupling under these conditions (Supplementary Figure 1), as compared with the normal pacing rate.

### 3.3. Conduction Velocity Is Correlated to Total Cell Na^+^Conductance and Gap Junctional Coupling

**Figure 4:**
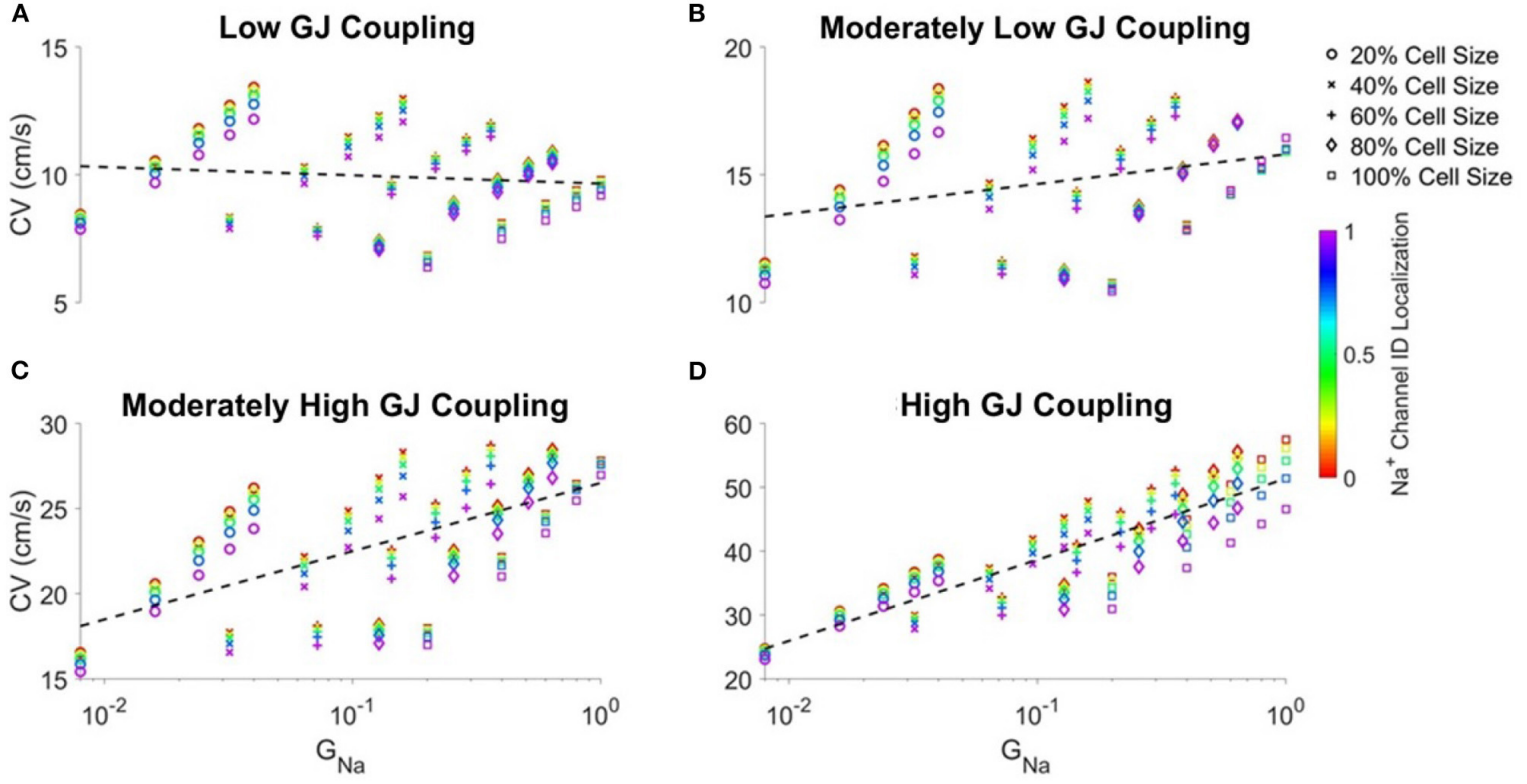
Correlation between conduction velocity (CV) and total cell Na^+^conductance (*G*_*Na*_) increases as gap junctional coupling increases. Conduction velocity (CV) is shown as a function of *G*_*Na*_ for different cell sizes and Na^+^ channel ID localization for **(A)** low, **(B)** moderately low, **(C)** moderately high, and **(D)** high GJ coupling. Pearson correlation coefficients *r* for low (*r* = −0.103), moderately low (*r* = 0.283), moderately high (*r* = 0.607), high (*r* = 0.865) GJ coupling. Parameters: Cleft width *w* = 20*nm*.

The previous analysis shows CV had a consistent positive dependence on Na^+^current density ρ_*Na*_, yet the relationship between CV and cell size depends on GJ coupling. As the total cell Na^+^conductance (*G*_*Na*_) depends on both cell size and Na^+^channel density, we next investigate how CV depends on *G*_*Na*_for varying GJ coupling conditions (Figure 4). For all GJ coupling strengths, we find discrete vertical “columns” of points, which correspond to the same *G*_*Na*_value (i.e., same ρ_*Na*_and *S*) and different values of Na^+^channel localization (*ID*_*Na*_). From these data, we observe that increased *ID*_*Na*_typically slows conduction, except for cases of moderately low GJ coupling and larger cell size. Additionally, we observe diagonal “bands” of points, which correspond with the same cell sizes and different ρ_*Na*_values, which illustrate that CV increases with increasing ρ_*Na*_, as in Figure 2.

With all of these dependencies within given “columns” or “bands,” we also consider if there is a distinct relationship between CV and *G*_*Na*_for a given GJ coupling strength. For low GJ coupling (Figure 4A), CV is overall very weakly correlated with *G*_*Na*_ (dashed black line), indicating that for these conditions, the total Na^+^channel conductance is not predictive of CV. However, as GJ coupling increases, the correlation between CV and *G*_*Na*_increases and approaches 1 for high GJ coupling (Figures 4B–D), demonstrating a closer relationship between conduction and total Na^+^current conductance.

**Figure 5:**
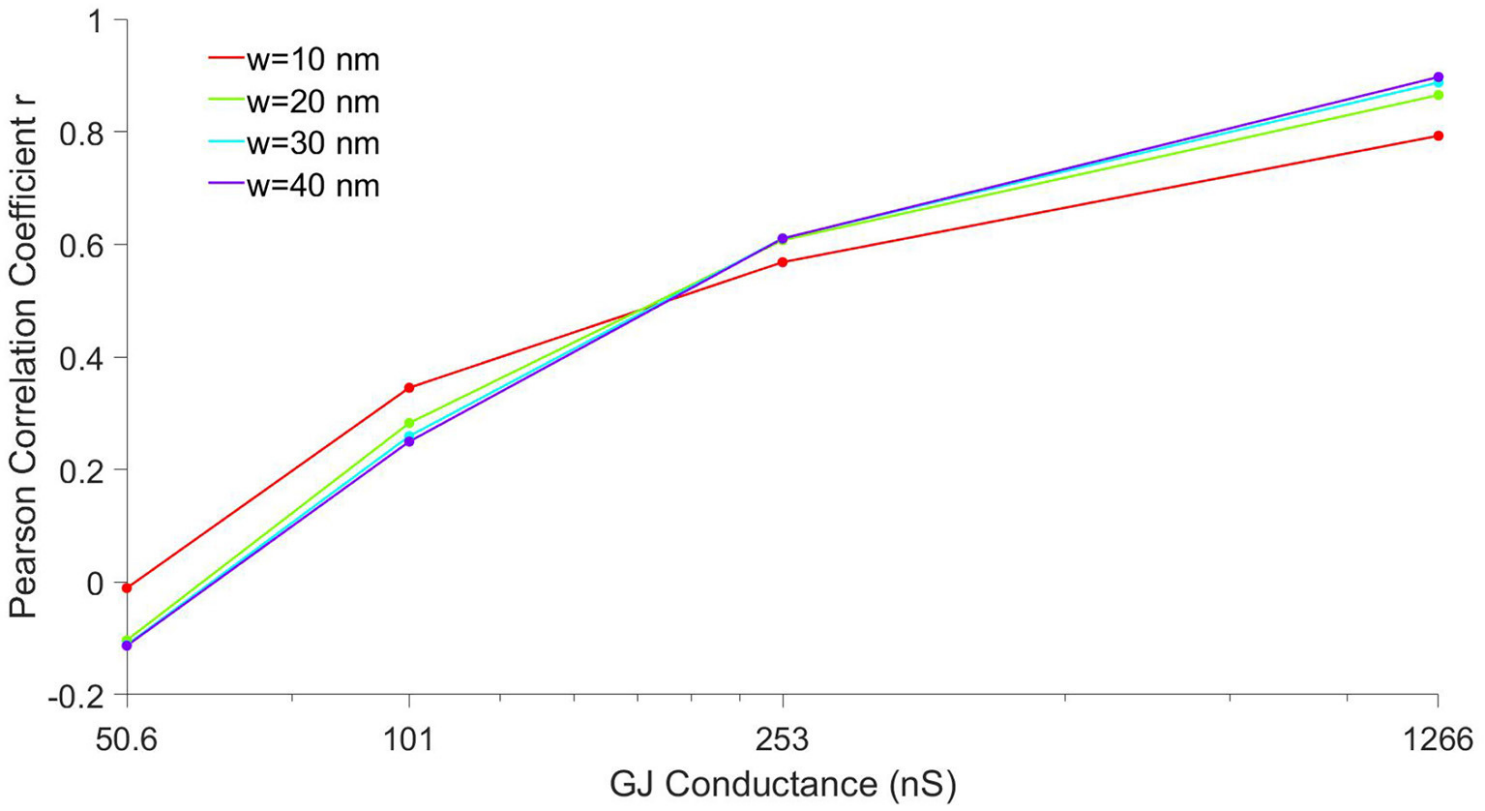
Correlation between conduction velocity (CV) and total cell Na^+^conductance depends on gap junctional coupling. The Pearson correlation coefficient *r* between CV and total cell Na^+^conductance (*G*_*Na*_) is shown as a function of GJ coupling conductance, for four different cleft width *w* values.

Prior work from us and others has shown that the intercellular width (*w*) is a critical parameter governing the strength of EpC effects and ultimately conduction (Kucera et al., 2002; Lin and Keener, 2010; Greer-Short et al., 2017; Nowak et al., 2020), so we next investigate how the correlation between CV and *G*_*Na*_depends on *w*. In Figure 5, we plot the Pearson correlation coefficient *r* as a function of GJ coupling for different values of *w*. For all cleft widths, the correlation increases from near 0 to near 1 as GJ coupling increases, as in Figure 4. However, for narrow clefts (red), the correlation is less sensitive to changes in GJ coupling, such that the correlation coefficient is more positive for lower GJ coupling and less positive for higher GJ coupling, compared with wider clefts. These results are consistent with EpC playing a larger role governing conduction for lower GJ coupling, such that there is a stronger relationship between CV and *G*_*Na*_for these conditions.

### 3.4. Conduction Velocity Depends on Developmental Stage

**Figure 6:**
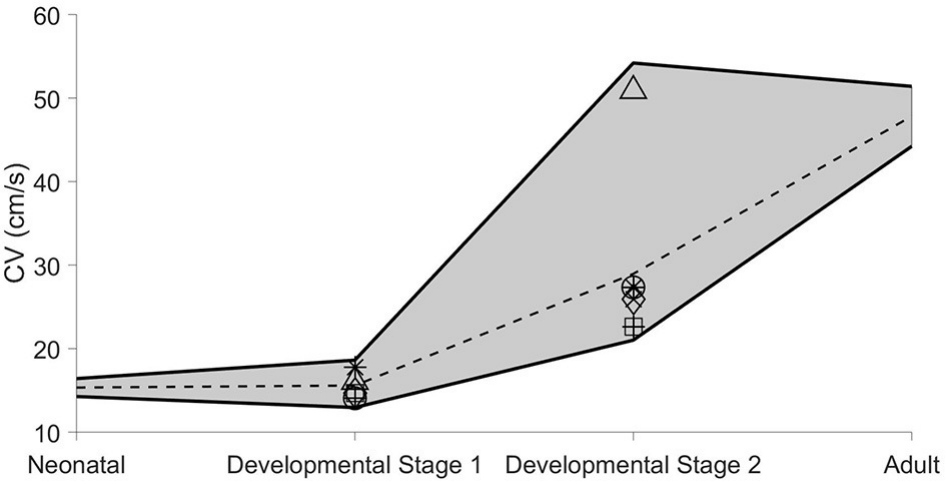
Age-associated ranges of conduction velocity (CV). The range of CV values (see text for details) are shown as functions of age-dependent progression. Parameter ranges and markers for each stage are shown in Supplementary Table 2. The dashed line shown for each stage represents the average CV value over all conditions within the specified parameter ranges. Cleft width *w* = 20*nm*.

The above results illustrate the complex relationship between conduction and cellular/tissue properties known to alter with age and development. As previously discussed, neonatal tissues are associated with low Na^+^channel expression and ID localization and smaller cell size, while adult tissues have larger cells and high Na^+^channel expression and localization at the ID. We hypothesize that, during both disease and development, these properties remain variable in both time and between patients. For a final analysis, we investigate CV for conditions representing an age-associated progression, considering neonatal, intermediate developmental stages, and adult tissue (Figure 6). To account for variability, we consider a range of values for each of the key parameters investigated throughout this study: cell size, Na^+^channel density, and Na^+^channel localization at the ID. These parameters were varied over a 20% range. The minimum and maximum CV values over all parameter conditions for each stage were plotted (solid black lines), along with the average over the different parameter values (dashed black line). Due to lack of evidence of the precise order in which these parameters change throughout the developmental process, we consider different possible parameter conditions for the intermediate stages: uniform ρ_*Na*_and *ID*_*Na*_increase, staged *S*and ρ_*Na*_increases, staged *S*and *ID*_*Na*_increases, and staged ρ_*Na*_and *ID*_*Na*_increases ranging from neonatal to adult tissue parameter values. The parameter ranges for each developmental stage are listed in Supplementary Table 2.

For the neonatal stage, the range of CVs is small and conduction speeds are slow. For the first intermediate developmental stage, the CV range is moderately larger, but the conduction remains slow. However, for the second intermediate developmental stage, the CV range is dramatically increased, ranging from roughly 20 cm/s to over 50 cm/s, suggesting that conduction greatly increases and can approach adult myocardium values, but may be more variable during this intermediate stage. For the adult stage, the range of CVs decreases, such that conduction is consistently within the normal faster propagation regime, suggesting robust conduction regardless of small changes in cellular and tissue properties.

## 4. Discussion

In this study, we investigate the regulation of key age-dependent properties, specifically cell size, Na^+^channel density and localization, and GJ coupling, on cardiac conduction. We summarize our key findings: CV is consistently increased by increased Na^+^current density, across all conditions. However, simulations predict that CV biphasically depends on cell size, depending on the strength of GJ coupling. That is, CV increases with increasing cell size for high GJ coupling, yet decreases with increasing cell size for low GJ coupling. As a consequence, CV and total cell Na^+^channel conductance are well-correlated in cardiac tissue with high GJ coupling, but not correlated with CV for low GJ coupling. We predict that the role of EpC governing conduction changes during development, such that neonatal tissue is less sensitive to changes in EpC due to smaller Na^+^channel ID localization. We postulate that even though GJ coupling is low during this early stage, the small cell size and therefore small membrane capacitance is such that the lower Na^+^current density is still sufficient to maintain robust conduction in the myocardium. However, conduction is very slow during this stage due to the relatively high ratio of cell-cell junctions for a given length of tissue. These findings are consistent with Swift et al. (2020), who found slower atrioventricular conduction in neonatal and early postnatal rats, compared with adult rat myocardium.

As development progresses, model predictions are consistent with faster conduction. Further, simulations predict that a wide range of conduction velocities are possible during intermediate developmental stages due to variability in cellular/tissue properties and the relative timing of developmental changes. However, despite similar variability in parameter values, this variability in CV prediction narrows in adult tissue and is consistent with experimental measures (George et al., 2019; King et al., 2021). Interestingly, we see a larger influence of EpC effects in larger cell sizes with reduced GJ coupling, as would be the case in intermediate developmental stages and in adult myocardium for pathological conditions such heart failure (Smith et al., 1991; Peters et al., 1997; Yao et al., 2003; Akar et al., 2004; Poelzing and Rosenbaum, 2004), indicating a possible mechanism for maintained conduction during such transitional or diseased states. Additionally, this enhanced variability in conduction for intermediate developmental stages may be desirable, as this variability suggests an ability to adapt and modulate cardiac activity in response to developmental perturbations, which inherently vary significantly for different individuals.

Previous studies have investigated the properties of Na^+^channels and GJs and their roles in the developing heart. Work by Harrell et al. (2007) found that Na_v_1.5mRNA was significantly more up-regulated in adult mouse hearts than in neonatal hearts. Similarly, Cordeiro et al. (2013) find that both peak and late *I*_*Na*_is significantly smaller in neonatal canine cardiomyocytes compared to that of adults, and Cai et al. (2011) found the same in human atrial cardiomyocytes. Vreeker et al. (2014) show that the GJ protein Cx43 transitions from diffusely distributed on the cardiomyocyte membrane to highly associated with mechanical junctions at the ID during development in human samples, as similarly shown in rat myocardium (Angst et al., 1997). In contrast, Peters et al. (1994) found that GJs and adherens junctions have a highly correlated distribution over postnatal development of the ventricle in human samples. Incorporating developmental changes in GJ distribution, Spach et al. (2000) previously simulated a neonatal cardiomyocyte with diffuse GJs and smaller size and found that conduction was slower in the neonatal tissue, compared with adult myocardium with GJs primarily localized at the ID and larger size.

While these studies are crucial in understanding cardiac development, to our knowledge, our study is the first to combine all these key developmental changes occurring in conjunction with each other, specifically cellular size, gap junctional conductance, and Na^+^channel expression and distribution. Thus, we find that ephaptic effects are more pronounced in larger cells with low GJ coupling. We also find that CV biphasically depends on cell size in a manner dependent on GJ coupling: CV is relatively fast for both low GJ coupling and small cell size and high GJ coupling and large cell size. Interestingly, we find that CV is correlated total Na^+^conductance for high GJ coupling. Finally, by incorporating previous data on developmental changes (Peters et al., 1994; Spach et al., 2000; Harrell et al., 2007; Cai et al., 2011; Cordeiro et al., 2013; Vreeker et al., 2014), we predict how the variability in the ranges for conduction change in conjunction with developmental stages.

Recently, we investigated the age-dependent manifestation of a long QT type 3 (LQT3)-associated gain-of-function mutation in Na_v_1.5 (Nowak et al., 2021). LQT3, while relatively rare, has a high mortality, reaching 49% (Vignati, 2007), and critically patients tend to remain asymptomatic until well after puberty (Beaufort-Krol et al., 2005; Wilde et al., 2016; Kutyifa et al., 2018). Interestingly, in pediatric patients with LQT syndromes, Na^+^channel blockers are commonly prescribed as a form of chronic management (Hanisch, 2001), and our results are consistent with the safety of this approach due to the weak dependence of conduction on overall total cell Na^+^channel conductance. In our study, we predicted that not only does the manifestation of arrhythmias depend on developmental stage, but that the sensitivity to changes in intercellular cleft width depended on these age-associated properties as well. Interestingly, Brugada syndrome (BrS), an inherited cardiac arrhythmia disorder caused a loss-of-function mutation in Na_v_1.5, also often manifests later in life; the average first event occurs around 42 years old (Milman et al., 2017). Our study is consistent with this clinical manifestation, as simulations predict that in these early developmental stages with low GJ coupling, conduction is less sensitive to overall Na^+^conductance (Figure 4), due to the overall small cell sizes in neonatal tissue.

Finally, we acknowledge limitations of our study. To study developmental changes in Na^+^channel distribution, we incorporate this critical subcellular detail in the representation of cardiac tissue; however, our model is still a simplification of the complex cardiac tissue structure. Specifically, our model assumes a simplified cylindrical cell and uniform intercellular cleft, while the geometry of individual cells and the ID structure is known to be complex heterogeneous (Veeraraghavan et al., 2015). Future work will focus on investigating how ID structure changes in development and impacts conduction (Moise et al., 2021). Additionally, cardiac tissue is a three-dimensional structure, and our one-dimensional cable representation cannot fully represent all aspects of developmental changes, such as GJ localization along the lateral membrane. In a two- or three-dimensional tissue, GJ redistribution from the lateral membrane to the ID would also be expected to impact conduction heterogeneously dependent on the direction of wavefront propagation relative to the underlying tissue geometry, such that early developmental stages are associated with isotropic conduction while anisotropic conduction is associated with adult myocardium, consistent with previous work from Spach et al. (2000). Further, while we observe minimal differences in CV between normal and slow pacing rates, Entz et al. (2016) previously showed that CV slowing at faster pacing rates differed between longitudinal and transverse conduction, and further was regulated by extracellular ionic composition, suggesting that properties governing EpC may similarly modulate heart rate dependence. Additionally, expression of ion channels vary across individual patients, and these differences may impact conduction during development. Further, recent work from the Posnack lab has shown that several expression of several key ion channel and calcium handling proteins vary with developmental stage in rats (Swift et al., 2020). We are particularly interested in similar changes in human myocardium, and future work will incorporate such details as available and established in the literature.

## Data Availability Statement

The raw data supporting the conclusions of this article will be made available by the authors, without undue reservation.

## Author Contributions

MN performed computational studies and wrote the original manuscript draft. RV contributed to the manuscript draft. SP and SW designed simulations, provided funding, and contributed to the manuscript draft. All authors contributed to the article and approved the submitted version.

## Funding

This study was supported by funding from the National Institutes of Health, grant numbers R01HL138003 (SW, SP), R01HL102298 (SP), and R01HL148736 (RV).

## Conflict of Interest

The authors declare that the research was conducted in the absence of any commercial or financial relationships that could be construed as a potential conflict of interest.

## Publisher's Note

All claims expressed in this article are solely those of the authors and do not necessarily represent those of their affiliated organizations, or those of the publisher, the editors and the reviewers. Any product that may be evaluated in this article, or claim that may be made by its manufacturer, is not guaranteed or endorsed by the publisher.
