# Supplementary material for: Cellular Size, Gap Junctions, and Sodium Channel Properties Govern Developmental Changes in Cardiac Conduction
Source: Front Physiol. 2021 Oct 25;12:731025. doi: 10.3389/fphys.2021.731025 (PMC8573326; doi:10.3389/fphys.2021.731025)
Supplement: Supplementary file 1 [file Data_Sheet_1.PDF]

Cellular size, gap junctions, and sodium channel properties govern developmental changes in cardiac conduction

SUPPORTING METHODS

Computational Model Details

The model for electrical conduction along a linear strand of 50 cells was modified from the ephaptic coupling model, as in our previous work (Greer-Short et al., 2017; Weinberg, 2017; Nowak et al., 2020, 2021) and as described by Kucera et al. (2002). The membrane of each cell was discretized into axial patches and 2 disc patches, one at each end of the cell (Fig. 1A). Each membrane patch generated currents proportional to the patch surface area: a capacitive current, with capacitance C_{ax} or C_{disc}, and ionic currents I_{ion} governed by the Luo-Rudy dynamic (LRd) guinea pig ventricular myocyte model (Livshitz and Rudy, 2007), modified as described below.

Intracellular nodes were connected by a myoplasmic resistance R_{myo}, and the end nodes between cells were connected by a gap junction resistance R_{gap}. The axial extracellular potentials (ϕ_e^{ax}) were assumed to be equal to 0, such that the axial intercellular potential (ϕ_i^{ax}) and axial transmembrane potential (V_m^{ax}) were equal. A T-shaped network of two axial resistances, each $\frac{1}{2}R_{cl}$, and a radial intercellular cleft resistance R_{radial}, connecting the intercellular cleft to the bulk extracellular space, accounted for electric field coupling in the intercellular cleft, such that the intercellular cleft and disc extracellular potentials, ϕ_e^{cleft} and ϕ_e^{disc}, respectively, may be nonzero. Electrical conduction parameters are given in Table S1. Equations for the resulting system of differential-algebraic equations for potentials at the intra- and extracellular nodes are given in our previous work (Weinberg, 2017).

In each simulation, a fixed percentage of sodium channels are defined to be localized at the intercalated disc, equally distributed between the membrane patches on the two cell ends, and the remaining sodium channels are distributed uniformly over the axial membrane patches. For example, if 90% of sodium channels are localized at the intercalated disc, i.e., the maximum I_{Na} conductance for each patch, $g_{Na,max}$, was set to 45% $\Sigma g_{Na,max}$ (total maximum conductance for the cell, which is fixed) in each disc patch, and the remaining 10% was distributed uniformly over the axial patches.

The formulation for LRd ionic currents, ionic concentration balances, and calcium handling were unchanged from Livshitz and Rudy (2007), except as noted below. The ionic current for each patch j is given by the sum of the Na^+, K^+, and Ca^{2+} currents carried by channels, pumps, and exchangers:

$$I^j_{ion} = I^j_{Na,tot} + I^j_{K,tot} + I^j_{Ca,tot}, \tag{S1}$$

where

$$I^j_{Na,tot} = I^j_{Na} + I^j_{Nab} + 3(I^j_{NCX} + I^j_{NCX,ss}) + I^j_{CaL,Na} + 3I^j_{NaK}, \tag{S2}$$

$$I^j_{K,tot} = I^j_{Kr} + I^j_{Ks} + I^j_{K1} + I^j_{Kp} + I^j_{CaL,K} - 2I^j_{NaK} + I^j_{stim}, \tag{S3}$$

$$I^j_{Ca,tot} = I^j_{CaL} + I^j_{Cab} + I^j_{pCa} + I^j_{CaT} - 2(I^j_{NCX} + I^j_{NCX,ss}). \tag{S4}$$

The currents are as follows: I_{Na} (fast Na^+ current), I_{Nab} (background Na^+ current), I_{NCX} (intracellular Na^+-Ca^{2+} exchanger current), $I_{NCX,ss}$ (subspace Na^+-Ca^{2+} exchanger current), $I_{CaL,Na}$ (Na^+ current carried by L-type Ca^{2+} channel), I_{NaK} (Na^+-K^+ pump current), I_{Kr} (rapid component of the delayed rectifier K^+ current), I_{Ks} (slow component of the delayed rectifier K^+ current), I_{K1} (inward rectifier K^+ current), I_{Kp} (plateau K^+ current), $I_{CaL,K}$ (K^+ current carried by L-type Ca^{2+} channel), I_{CaL} (Ca^{2+} current carried by the L-type Ca^{2+} channel), I_{Cab} (background Ca^{2+} current), I_{pCa} (Ca^{2+} pump current), I_{CaT} (T-type Ca^{2+} current), and I_{stim} (stimulus current).

The Hodgkin-Huxley type gating model for the I_{Na} current was replaced with a 13-state Markov chain formulation developed by Clancy et al. (2002), to reproduce a wild-type $Na_v1.5$ channel.

In addition to accounting for the electric field effects via the intercellular cleft extracellular potential, we also accounted for localized depletion of intercellular cleft [Na^+]. The Na^+ reversal potential at the intercalated disc membrane patches was given by

$$E_{Na}^{disc} = \frac{RT}{z_{Na}F} \ln\left(\frac{[Na^+]_e^{cleft}}{[Na^+]_i^{disc}}\right), \tag{S5}$$

where $z_{Na} = 1$ is the sodium ion valence, *F* is Faraday's constant, *R* is the gas constant, and *T* is the absolute temperature.

The extracellular sodium concentrations in the clefts between cells ($[Na^+]_e^{cleft}$) was governed by

$$\frac{d[Na^+]_e^{cleft}}{dt} = \frac{A_d \sum I_{Na}^{disc}}{z_{Na}FV_{cleft}} + \frac{[Na^+]_e^{bulk} - [Na^+]_e^{cleft}}{\tau_{Na}^{cleft}}, \tag{S6}$$

where the bulk extracellular sodium concentration ($[Na^+]_e^{bulk}$) is fixed at 140 mM, $A_d = \pi r^2$ is the disc patch area, r is the cell radius, $\sum I_{Na}^{disc}$ is the sum of I_{Na} from the disc membrane patches, $V_{cleft} = \Theta_{cleft} \cdot w \cdot \pi r^2$ is the nanodomain cleft volume, $\Theta_{cleft} = 0.1$ accounts for the cleft volume fraction that comprises the Na^+ nanodomains localized at the intercalated disc, and $\tau_{Na}^{cleft} = 303$ ms is the time constant for cleft refilling.

The LRd model accounts for dynamic changes in the ionic concentrations of intracellular Na^+, K^+, and Ca^{2+} in the axial and disc compartments. The dynamics of the intracellular concentration of ion X in compartment *j* are governed by

$$\frac{d[X]_i^j}{dt} = -\frac{A_j I_{X,tot}^j}{zFV_j} + \frac{[X]_i^{j-1} - [X]_i^j}{\tau_X} + \frac{[X]_i^{j-1} - [X]_i^j}{\tau_X}, \tag{S7}$$

where A_j is the compartment surface area, $I_{X,tot}^j$ is the total sum of currents carried by ion X, V_j is the volume associated with compartment *j* ($V_j = L_p \cdot \pi r^2$ for axial compartment, and $V_j = L_p \cdot \pi r^2/2$ for disc compartments), and τ_X is the time constant for transfer between compartments. No intercellular ionic flux were assumed. Time constants are given by $\tau_X = L_p^2/(2D_X)$, where diffusion coefficients (in units of $\mu m^2\ ms^{-1}$) are $D_{Na} = 0.5$ (Despa and Bers, 2003), $D_K = 1.3$ (Hodgkin and Keynes, 1953), $D_{Ca} = 0.25$ (Smith, 2005), and $D_{Cl} = 0.6$ (estimated to be equal to Na^+), such that (in units of ms) $\tau_{Na} = 83.3$, $\tau_K = 38.5$, $\tau_{Ca} = 200$, and $\tau_{Cl} = 83.3$.

A subspace compartment was diffusively coupled to each intracellular compartment, containing Na^+ and Ca^{2+}. Ca^{2+} influx via L-type calcium channels and 20% of Na^+-Ca^{2+} exchange (NCX) occurs in the subspace compartments. We assumed that NCX and Na^+-K^+ ATPase (NaK) activity is uniformly distributed between the axial compartments and not present at the disc. The equations governing the dynamic of subspace [Na^+] and [Ca^{2+}], and [Ca^{2+}] in network and junctional sarcoplasmic reticulum, NSR and JSR, respectively, included transfer flux terms between adjacent subspace compartments, with time constant given by τ_{Ca}. The equations governing subspace [Na^+] and [Ca^{2+}] also included transfer flux between the subspace and the corresponding intracellular compartment, with time constant of 0.2 ms (Decker et al., 2009).

To match experimental measurements for conduction velocity , the maximum conductances for I_{Na} and I_{CaL} were scaled by factors of 2.4 and 1.4, respectively. To maintain calcium homeostasis, L-type calcium current steady-state activation curve was shifted -2 mV, JSR release half-saturation constant K_{rel}^{ss} was increased from 1 to 1.5 mM, and the release time constant was increased by 50%.

Numerical integration and analysis

We applied a numerical integration scheme similar to Kucera et al. (2002). At each time point *t*, we calculated the membrane current for each patch, based on the membrane patch potentials V_m and gating variable values. By applying Kirchhoff's current law at every node and assuming that the membrane currents do not change over a small time interval $\Delta t_1 = 0.5\ \mu s$, i.e., between time *t* and $t + \Delta t_1$, we obtained a system of coupled first-order differential equations and algebraic relationships. We numerically integrated this system using a forward Euler method, with time step $\Delta t_2 = \Delta t_1/10 = 0.05\ \mu s$, and solved for all intracellular and extracellular potentials until time $t + \Delta t_1$. Gating variables and ionic concentrations were numerically integrated between time *t* and $t + \Delta t_1$ using a forward Euler method, with time step Δt_1. Using values for the membrane patch potentials V_m and gating variables, membrane currents were then recomputed at time $t + \Delta t_1$.

Initial conditions for each cell in the myocyte strand were established by simulating the single cell for 100 beats at a given pacing rate. Propagating electrical waves were initiated by applying a 0.5-ms stimulus current of amplitude $-800\mu A/cm^2$ in the two center axial nodes of cells 1 to 5. Myocyte strands were first paced to a steady-state and then simulations were run for an additional 10 beats for CV measurements. CV was computed by linear regression of the activation times of cells 15 to 35.

SUPPORTING FIGURE

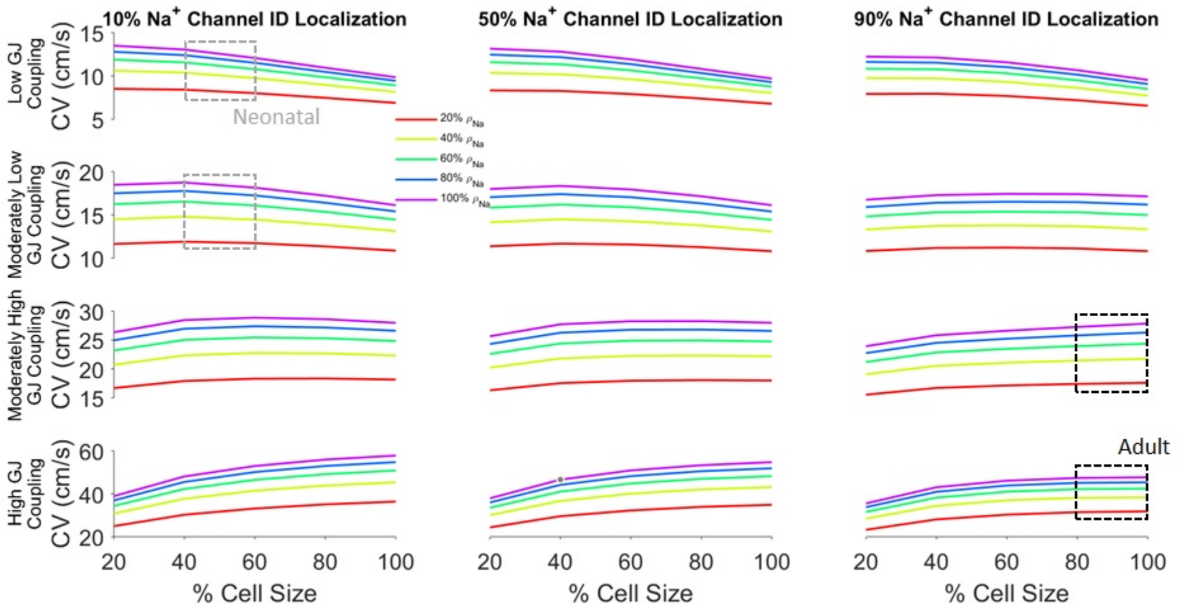

Figure S1: Conduction velocity (CV) depends on key cellular and tissue properties for slower pacing rate. CV is shown as a function of cell size for different values of Na^+channel densities (ρ_{Na}) for low (A), moderately low (B), moderately high (C), and high (D) GJ coupling and 10% (*left*), 50% (*middle*), and 90% (*right*) Na^+ channel ID localization (ID_{Na}). Parameters: Cleft width $w = 20\ nm$. BCL $= 1000$ ms. Parameter regimes associated with neonatal (gray boxes) and adult (black boxes) tissue are highlighted.

SUPPORTING TABLES

Variable	Name	Value
C_m	Specific Membrane Capacitance	10^{-8} μF/μm^2
L_p	Axial Patch Length	10 μm
$L = Sn_p^0 L_p$	Cell Length	Varied (depends on S)
$r = Sr_0$	Cell Radius	Varied (depends on S)
$C_{ax} = 2\pi r L_p \cdot C_m$	Axial Patch Capacitance	Varied (depends on S)
$C_{disc} = \pi r^2 \cdot C_m$	Disc Patch Capacitance	Varied (depends on S)
ρ_{myo}	Myoplasmic Resistivity	$150\ \Omega \cdot \text{cm}$
ρ_{ext}	Extracellular Resistivity	$150\ \Omega \cdot \text{cm}$
$R_{myo} = \rho_{myo} \cdot L_p/(\pi r^2)$	Myoplasmic Resistance	$39.45\ \text{k}\Omega$
$R_{gap} = R_{gap}^0/f_{gap}$	Gap Junctional Resistance	Varied (depends on f_{gap})
$R_{radial} = \rho_{ext}/(8\pi w)$	Radial Intercellular Cleft Resistance	Varied (depends on w)
$R_{cl} = \rho_{ext} \cdot w/(\pi r^2)$	Axial Intercellular Cleft Resistance	Varied (depends on w)

Table S1. Electrical parameters and equations from Kucera et al. Kucera et al. (2002) ephaptic coupling model, including parameters dependent on cell size (S), relative gap junctional conductance (f_{gap}), and cleft width (w) as described in the main text.

Development stage	Cell size (S)	Na^+ channel density (ρ_{Na})	Na^+ channel ID localization (ID_{Na})	Gap junctional coupling (nS)
**Neonatal:**	40-60	40-60	10-30	101
**Intermediate Stage 1**				
Uniform ρ_{Na}, ID_{Na} increases △:	60-80	60-80	30-50	101
Staged S, ρ_{Na} increase ○:	80-100	40-60	10-30	101
Staged S, ID_{Na} increase +:	80-100	40-60	10-30	101
Staged ρ_{Na}, S increase ✳:	40-60	80-100	10-30	101
Staged ρ_{Na}, ID_{Na} increase ×:	40-60	80-100	10-30	101
Staged ID_{Na}, S increase □:	40-60	40-60	70-90	101
Staged ID_{Na}, ρ_{Na} increase ◇:	40-60	40-60	70-90	101
**Intermediate Stage 2**				
Uniform ρ_{Na}, ID_{Na} increases △:	80-100	80-100	50-70	253
Staged S, ρ_{Na} increases ○:	80-100	80-100	10-30	253
Staged S, ID_{Na} increases +:	80-100	40-60	70-90	253
Staged ρ_{Na}, S increases ✳:	80-100	80-100	10-30	253
Staged ρ_{Na}, ID_{Na} increases ×:	40-60	80-100	70-90	253
Staged ID_{Na}, S increases □:	80-100	40-60	70-90	253
Staged ID_{Na}, ρ_{Na} increases ◇:	40-60	80-100	70-90	253
**Adult:**	80-100	80-100	70-90	1266

Table S2. Range of model parameters for age progression in Fig. 6. Values given as a percentage (%), as described in the Methods. Values for cell size are estimated based neonatal and adult myocyte measurements of membrane capacitance, surface area, and cell volume [Cordeiro et al. (2013); Vreeker et al. (2014); Spach et al. (2000)].

REFERENCES

Clancy, C. E., Tateyama, M., Kass, R. S., et al. (2002). Insights into the molecular mechanisms of bradycardia-triggered arrhythmias in long qt-3 syndrome. The Journal of clinical investigation 110, 1251–1262

Cordeiro, J. M., Panama, B. K., Goodrow, R., Zygmunt, A. C., White, C., Treat, J. A., et al. (2013). Developmental changes in expression and biophysics of ion channels in the canine ventricle. Journal of molecular and cellular cardiology 64, 79–89

Decker, K. F., Heijman, J., Silva, J. R., Hund, T. J., and Rudy, Y. (2009). Properties and ionic mechanisms of action potential adaptation, restitution, and accommodation in canine epicardium. American Journal of Physiology-Heart and Circulatory Physiology 296, H1017–H1026

Despa, S. and Bers, D. M. (2003). Na/k pump current and [na] i in rabbit ventricular myocytes: local [na] i depletion and na buffering. Biophysical journal 84, 4157–4166

Greer-Short, A., George, S. A., Poelzing, S., and Weinberg, S. H. (2017). Revealing the concealed nature of long-qt type 3 syndrome. Circulation: Arrhythmia and Electrophysiology 10, e004400

Hodgkin, A. and Keynes, R. (1953). The mobility and diffusion coefficient of potassium in giant axons from sepia. The Journal of physiology 119, 513–528

Kucera, J. P., Rohr, S., and Rudy, Y. (2002). Localization of sodium channels in intercalated disks modulates cardiac conduction. Circulation research 91, 1176–1182

Livshitz, L. M. and Rudy, Y. (2007). Regulation of ca2+ and electrical alternans in cardiac myocytes: role of camkii and repolarizing currents. American Journal of Physiology-Heart and Circulatory Physiology 292, H2854–H2866

Nowak, M. B., Greer-Short, A., Wan, X., Wu, X., Deschênes, I., Weinberg, S. H., et al. (2020). Intercellular sodium regulates repolarization in cardiac tissue with sodium channel gain-of-function. Biophysical Journal

Nowak, M. B., Poelzing, S., and Weinberg, S. H. (2021). Mechanisms underlying age-associated manifestation of cardiac sodium channel gain-of-function. Journal of molecular and cellular cardiology 153, 60–71

Smith, G. D. (2005). Modeling Intracellular Calcium: Diffusion, Dynamics, and Domains (Taylor & Francis Group). 339–374

Spach, M. S., Heidlage, J. F., Dolber, P. C., and Barr, R. C. (2000). Electrophysiological effects of remodeling cardiac gap junctions and cell size: experimental and model studies of normal cardiac growth. Circulation research 86, 302–311

Vreeker, A., Van Stuijvenberg, L., Hund, T. J., Mohler, P. J., Nikkels, P. G., and Van Veen, T. A. (2014). Assembly of the cardiac intercalated disk during pre-and postnatal development of the human heart. PloS one 9

Weinberg, S. (2017). Ephaptic coupling rescues conduction failure in weakly coupled cardiac tissue with voltage-gated gap junctions. Chaos: An Interdisciplinary Journal of Nonlinear Science 27, 093908
